# Misperception of Visual Verticality in Patients with Primary Headache Disorders: A Systematic Review with Meta-Analysis

**DOI:** 10.3390/brainsci10100664

**Published:** 2020-09-24

**Authors:** Esteban Obrero-Gaitán, María Manrique-Navarro, Miguel Ángel Lérida-Ortega, Daniel Rodríguez-Almagro, María Catalina Osuna-Pérez, Rafael Lomas-Vega

**Affiliations:** 1 Department of Health Sciences, University of Jaen, 23071 Jaen, Spain; eobrero@ujaen.es (E.O.-G.); malerida@ujaen.es (M.Á.L.-O.); mcosuna@ujaen.es (M.C.O.-P.); rlomas@ujaen.es (R.L.-V.); 2 Centro de Fisioterapia “Pedro Chueca FisioTec S.A.”, 28015 Madrid, Spain; maria.manrique.navarro@gmail.com; 3 Hospital San Agustín de Linares, 23700 Linares, Spain; 4 Escuela de Osteopatía de Madrid S.L., 28807 Alcalá de Henares, Madrid, Spain

**Keywords:** primary headache disorders, migraine, tension-type headache, perception of visual verticality, subjective visual vertical, sensory integration

## Abstract

Migraine and tension-type headache (TTH) are the two most prevalent primary headache disorders (PHDs) that may involve visual and vestibular impairments, neck pain, and postural unsteadiness. The perception of visual verticality (VV) has been studied in patients diagnosed with PHD to assess balance disorders showing varying findings. Our study aimed to assess the VV perception in patients diagnosed with PHD in comparison to healthy controls. A systematic review with meta-analysis was carried out in PubMed MEDLINE, Scopus, WOS, CINAHL, and SciELO. The Cohen standardized mean difference (SMD) was used to estimate the differences between exposed and healthy controls. Seven studies with 816 participants were included. The quality of included studies, according to the Newcastle–Ottawa Scale (NOS), was moderate (mean score of 5.2). Patients diagnosed with PHD showed a moderate misperception of VV as assessed with the subjective visual vertical (SVV) test (SMD = 0.530; 95% CI = 0.225, 0.836; *p* < 0.001). Specifically, a misperception of the SVV was found in patients with migraine (SMD = 0.369; 95% CI = 0.1, 0.638; *p* = 0.007) and with TTH (SMD = 1.122; 95% CI = 0.540, 1.704; *p* < 0.001). This review shows a misperception of VV in patients with migraine and TTH when assessed with the SVV test, being higher in patients with TTH, although the THH sample size was low.

## 1. Introduction

Primary headache disorders (PHDs) are a significant worldwide public health problem [1]. Although not a direct cause of mortality, headaches are responsible for a greater number of years lived with disability than the rest of neurologic diseases combined [2]. Within these disorders, migraine and tension-type headache (TTH) are the most prevalent, and are the third and sixth disorders with the highest prevalence at a global level [1]. Their joint global prevalence is estimated at 40.5% [1], mainly affecting women, students, and residents of large cities [3]. Both migraine and TTH are associated with an enormous individual impact with a large influence at the socioeconomic level, generating high associated costs [4].

It has been possible to observe how certain alterations frequently present in conjunction with headache. In particular, comorbidity between neck pain and headache disorders has been widely assessed in numerous studies [5,6,7,8]. Although the physio-pathological processes of both migraine and TTH are not well known, it is known that cervical structures play an important role in these processes [9,10,11,12]. Moreover, the visual system is also involved in the development of PHD. In this regard, not only has a reduction in the speed of smooth pursuit and saccadic eye movements been observed, but also an affectation of binocular coordination during smooth pursuit movements in patients with TTH during continuous visual efforts maintained under inappropriate conditions [13]. Furthermore, vestibular dysfunction and non-specific balance disturbances have also been widely linked to headaches, despite the origin of their concomitance being unknown [14,15,16]. It is possible that a sensitization process at the trigeminal level may be the link that allows observation of the concomitance previously mentioned [9,17,18].

**Figure 1:**
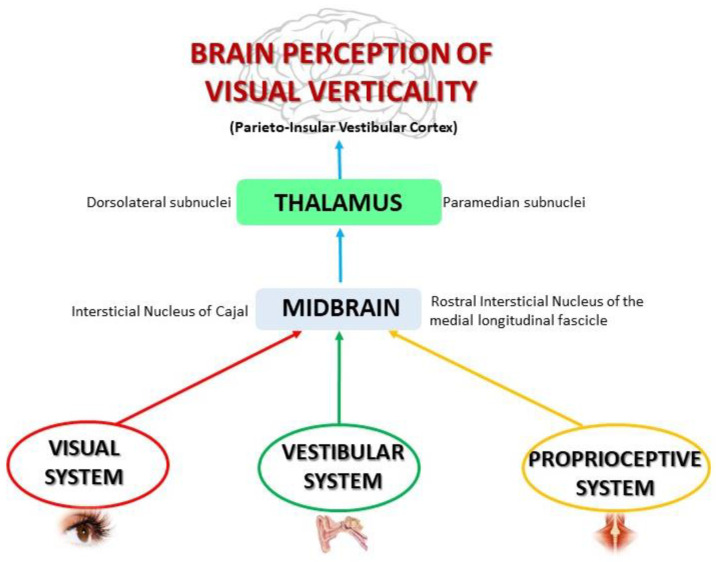
Visual, vestibular, and proprioceptive contributions to build the sense of verticality.

The representation of vertical at the central level makes control of spatial orientation and body stabilization processes possible [19]. Visual verticality (VV) perception is substantiated by a gravitational input that is processed by the central nervous system (CNS) and is shaped by vestibular, visual, and somatosensory information [19,20] (Figure 1). Vestibular and visual information lead to cognizance of vertical self-position in relation to the environment, while the somatosensory system informs about vertical self-position in relation to gravity force [21].

The perception of verticality can be assessed through the estimation of one’s own body verticality (subjective postural vertical, or SPV), the estimation of the verticality of objects that appear in the visual field (subjective visual vertical test, SVV; rod and frame test, RFT) or by touch (haptic vertical) [19]. The main test proposed to assess perception of verticality in both research and clinical practice is the SVV test [22]. In the SVV test, patients have to align a visible luminous line, in darkness, with the perfect vertical (those marked by gravity) without other visual references [23,24,25]. This test also allows the assessment of one’s own body position in space with respect to gravity [26]. Another test designed to determine the perception of VV is the RFT [27,28]. Although both tests evaluate the visual perception of verticality, the SVV mainly assesses the vestibular contribution to the creation of the sense of verticality [29], while the RFT mainly assesses proprioceptive and visual inputs [30].

Due to the relationship observed among vestibular, visual, and somatosensory alterations in patients with primary headaches, it could be possible to deduce a possible disturbance in vestibular, visual, or somatosensory inputs in patients with headache. Thus, patients with headache could misperceive verticality because of the important role that these three systems of information play in the formation of the central tridimensional pattern of verticality. Different studies have analyzed the visual and vestibular contribution to the sense of verticality in patients with migraine and TTH with varying results. Differences may be able to be explained by methodological differences in the use of the SVV test, or the correct diagnosis of patients with PHD. In light of the differing results of previous studies and the absence of reviews on the subject, our systematic review with meta-analysis aimed to collate the available evidence and analyze the perception of VV in patients with PHD compared to healthy subjects.

## 2. Materials and Methods

### 2.1. Protocol Review

To perform this meta-analysis, authors have followed the recommendations proposed in the Preferred Reporting Items for Systematic Reviews and Meta-Analyses (PRISMA) [31] statement and the suggestions of the Meta-Analysis of Observational Studies in Epidemiology (MOOSE) group [32].

### 2.2. Data Sources and Search Strategy

The search strategy was performed in PubMed, Scopus, Web of Science, CINAHL, and SciELO by two authors (E.O.-G. and R.L.-V.), independently, between 1 February 2020 and 30 March 2020. The authors also examined reference lists from retrieved full-text studies, guidelines, and reviews. The keywords used in the bibliographic search strategy were “headache disorders, primary,” “migraine disorders,” “tension-type headache,” “subjective visual vertical,” and “perception of visual verticality.” Medical Subject Headings (MeSH), EBSCOhost, and the main keywords found in previous published studies were chosen as keywords. According to the database, a specific keyword combination was employed using the appropriate tags and the Boolean operators “and” or “not”. No publication date and language filters were used. In Table 1, the authors summarize the search strategy used to identify the studies in the health databases.

### 2.3. Study Selection and Inclusion Criteria

Studies reported with the search strategy for possible inclusion were assessed in an independent screening for each of the two blinded authors who performed this phase (E.O.-G and R.L.-V). Irrelevant studies were excluded based on the screening of the title and abstract. Each reference was assessed in detail if at least one of the researchers selected it during the title and abstract review. Each reviewer re-evaluated the discrepant studies, and disagreements were resolved by consultation with a third researcher (D.R.-A.) during the full-text review.

The inclusion criteria applied were: (1) observational studies (including cross-sectional, cohort, and case–control studies); (2) studies that assessed the ability of patients with migraine or TTH to estimate the perception of VV; (3) studies that used the static SVV test to estimate VV perception; and (4) studies that included a comparison group of healthy controls. The exclusion criteria used were: (1) observational studies with only one group; (2) studies that assessed the perception of VV after any physical or pharmacological intervention; (3) studies that did not report mean or standard deviation of the SVV estimation; and (4) studies that were not susceptible to obtain the absolute error mean and its standard deviation according to validated procedures [33,34].

### 2.4. Data Extraction

Data of the exposed and non-exposed groups of each study were collected by two reviewers (M.M.-N and M.C.O.-P.) independently, employing a standardized data collection form. Discrepancies were resolved with the intervention of a third author (R.L.-V.).

The characteristics collected of each included study were the authorship, publication date, research design, sample size, number of participants in the exposed (patients diagnosed with PHD) and non-exposed group (healthy subjects), age of participants classified in two age groups (adult: 19–44 years old and middle aged: 45–64 years old), gender sex (female or male), type of PHD (migraine or TTH), and phase of progression (acute, subacute, or chronic). The primary outcome measure was the perception of VV obtained in each group using the SVV test. All data included in our analysis according the SVV were obtained without any therapeutical intervention in the control or exposed groups. We obtained the body position, the head position (fixed or non-fixed), and the used SVV test. Following the recommendations of the *Cochrane Handbook for Systematic Reviews of Interventions* [33] and Hozo et al. (2005) [34], studies using statistics different from the mean or SD susceptible to be selected for the quantitative synthesis were also included.

### 2.5. Quality Assessment

To evaluate the quality of the studies included in this meta-analysis, the Newcastle–Ottawa scale (NOS) was applied [35]. The domains explored by this scale are: “selection of study groups” (maximum, 4 stars), “comparability of groups” (maximum, 2 stars), and “ascertainment of exposure/outcome” (maximum, 3 stars) [36]. Quality scores ranged from 0 (lowest) to 9 stars (highest) [37]. The quality classification of the included studies according to NOS score is: low (score 1 to 3), medium (score 4 to 6), and high quality (score 7 to 9) [37]. Inconsistency and imprecision were assessed according to Meader et al. (2014) [38] and the Grading of Recommendations Assessment, Development and Evaluation (GRADE) system [38]. Inconsistency was evaluated through heterogeneity of findings in individual studies [39] and imprecision through the number of included studies (large: >10 studies, medium: 5 to 10 studies, and small: <5 studies) and with the median sample size of each study (high: >300 subjects, medium: 100 to 300 subjects, and low: <100 subjects) [33,38]. The assessment of publication bias risk was detailed in statistical analysis.

This process was performed independently by two authors (D.R.-A. and M.Á.L.-O.) who assessed the quality of the included studies. The disagreements that arose were resolved by a third author (R.L.-V.).

### 2.6. Statistical Analysis

The Comprehensive Meta-Analysis 3.3.070 (Biostat, Englewood, NJ, USA) was used to carry out the meta-analysis [40]. Two authors designed and conducted the statistical analysis (E.O.-G and R.L.-V.). Based on the suggestions of Cooper et al. (2009) [41], we employed a random-effects model of DerSimonian and Laird to estimate the pooled effect and its 95% confidence interval (95% CI) [42] to generalize the study’s findings. The Cohen’s standardized mean difference (SMD) was selected to calculate the pooled effect [43]. The SMD can be interpreted as small (SMD = 0.2), moderate (SMD = 0.5), or large (SMD > 0.8) [44]. Forest plots were used to display our findings [45]. The risk of publication bias was assessed with the visualization of the funnel plot [46,47] (symmetric = low risk of publication bias or asymmetric = high risk of publication bias). In addition, we used the trim-and-fill method [48] to estimate the adjusted pooled effect, considering a possible publication bias [49]. Heterogeneity was assessed using Cochran’s Q test [50] and the *I*^2^ Statistic of Higgins (<25% indicates low heterogeneity; 25–50% moderate heterogeneity; and >50% large heterogeneity) and *p* < 0.1 indicated large heterogeneity [39,51]. We performed a general meta-analysis to assess the perception of VV in all PHDs, and finally, we assessed the perception of VV in two different meta-analyses according to the type of PHD (migraine or TTH).

### 2.7. Additional Analysis

A sensitivity analysis (using the leave-one-out method) was performed to assess the contribution of each study to the pooled estimate in the meta-analysis [41].

### 2.8. Subgroup Analysis

Subgroup analysis was made according to the type of PHD (migraine or TTH), the position of the head in the SVV measure (fixed or non-fixed head position), and the SVV test used (bucket test or rod projected, both in darkness).

## 3. Results

### 3.1. Study Selection

**Figure 2:**
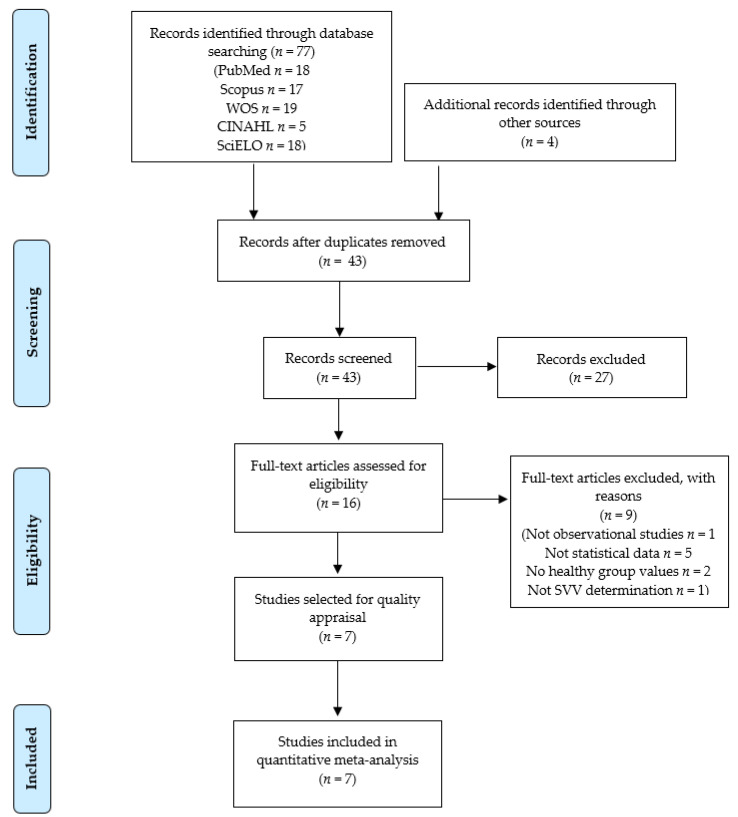
Preferred Reporting Items for Systematic Reviews and Meta-Analyses (PRISMA) flow chart.

The PRISMA flow chart in Figure 1 summarizes the bibliographic search and study selection process. Based on the search criteria, 77 references were retrieved from the different databases, plus four additional records, which were recuperated after reviewing the reference lists of other studies. After removing duplicates, 43 studies were reviewed by title/abstract. Twenty-seven references were excluded as they were not relevant, and nine studies were removed for not meeting the inclusion criteria. Figure 2 shows the number of records removed and the reasons for exclusion. Finally, seven studies [52,53,54,55,56,57,58] were included in the present review.

### 3.2. Characteristics of the Seven Studies Included in the Meta-Analysis

The main characteristics of the studies included in the review appears in Table 2. The seven studies included in this review comprised 10 samples with 10 independent comparisons providing data from 816 participants (34% males and 66% females). The seven studies [52,53,54,55,56,57,58] had eight independent comparisons, including 669 patients with migraine (mean age of 36.72 ± 4.9 years old); two of the studies [52,56] had two independent comparisons with reported data for 147 subjects with TTH (mean age of 39.52 ± 6.81 years old). The exposed group incorporated patients with migraine and TTH, and 364 healthy subjects formed the non-exposed group (mean age 35.87 ± 5.81 years old). All subjects included in this meta-analysis were in the chronic phase of the disease. The criteria of diagnosis of PHD in the included studies were the The International Classification of Headache Disorders [52,55,56,58], the International Classification of Vestibular Disorders [54,57], and the Neuhauster’s Classification [53]. The chosen measure to assess the perception of VV was the SVV test in the sitting position. Two studies assessed the SVV with the bucket test [54,56] and the rest of the studies analyzed the SVV with techniques based on the projection of a fluorescent rod on a screen that was oriented to the earth vertical with a manual controller (joystick potentiometer) [52,53,55,57,58]. More characteristics about the SVV measurement in each study are shown in Table 3.

### 3.3. Quality Assessment of the Studies Included in the Meta-Analysis

The methodological quality of the studies included in this review, as evaluated with the Newcastle–Ottawa Scale (NOS), was moderate (NOS mean score of 6.4). Four studies [52,54,55,58] (57% of included studies) showed a medium quality, and three studies showed a high quality [53,56,57] (47% of the total). Table 4 summarizes the NOS rating for selection, comparability, and exposure/outcome of the selected studies.

### 3.4. Results of the Overall Meta-Analysis on Perception of Visual Verticality in Patients with Primary Headache Disorders

**Figure 3:**
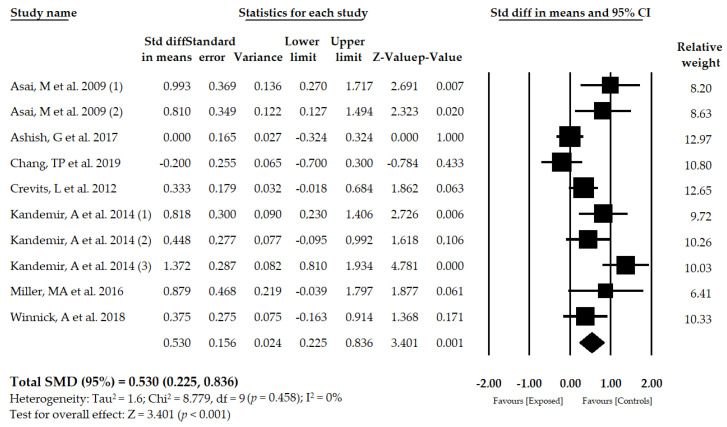
Forest plot for the perception of visual verticality, assessed with the SVV test, in patients with primary headache disorders.

Seven studies [52,53,54,55,56,57,58] including 10 samples with 10 independent comparisons reported data for 816 patients with PHD (mean age of 37.27 ± 5.1 years old) in which perception of VV was assessed with the SVV test in the sitting position. The mean deviation of the SVV from the true vertical was moderate in exposed subjects compared to the group of healthy controls (SMD = 0.530; 95% CI = 0.225, 0.836; *p* < 0.001) (Figure 3, Table 5, Supplementary Figure S1). The funnel plot is slightly asymmetric (Figure S2), suggesting a possible risk of publication bias. The trim-and-fill method was used to adjust the pooled effect considering the possible publication bias (adjusted SMD = 0.335), which varied by 37%. Heterogeneity was not present in this meta-analysis (*I*^2^ = 0%, *p* = 0.458), and the number of participants per study was 81.6, showing a low level of precision in our findings. The sensitivity analysis (leave-one-out method) yielded a pooled estimate that varied 22% when compared to the original pooled estimate.

### 3.5. Results of the Meta-Analysis in Patients with Migraine

**Figure 4:**
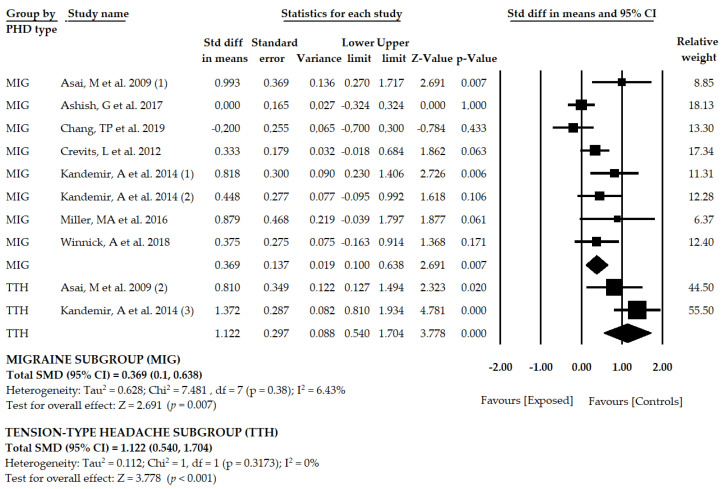
Forest plot for the perception of visual verticality, assessed with the SVV test, in subgroups of patients with migraine or tension-type headache.

Seven studies [52,53,54,55,56,57,58] including eight samples and eight independent comparisons reported data for 669 participants with migraine, with a mean age of 36.72 ± 4.9 years old. Our findings showed that the mean deviation of the SVV was moderate–low in patients with migraine in comparison to healthy subjects (SMD = 0.369; 95% CI = 0.1, 0.638; *p* = 0.007) (Table 5, Figure 4, Supplementary Figure S1). The funnel plot was slightly asymmetric (Figure S3), and the adjusted pooled effect, using the trim-and-fill method (adjusted SMD = 0.267), suggested a possible risk of publication bias. Heterogeneity in this meta-analysis was very low (*I*^2^ = 6.4%, *p* = 0.38), and the precision level was also low (83.62 participants per study). Sensitivity analysis (one study removed) yielded pooled estimates of 21% with respect to the original pooled estimate.

### 3.6. Results of the Meta-Analysis in Patients with Tension-Type Headache

Two studies [52,56] with two samples and two independent comparisons reported data for 147 subjects with TTH (mean age of 39.52 ± 6.81 years old). The SVV test was used to assess the perception of verticality in these patients and showed a large deviation of the SVV in participants with TTH with respect to the non-exposed group (SMD = 1.122; CI 95% = 0.540, 1.704; *p* < 0.001) (Table 5, Figure 4, Supplementary Figure S1). Risk of publication bias was not calculated due to the low number of included studies. Heterogeneity was not present in this meta-analysis (*I*^2^ = 0%, *p* = 0.3173). The precision level was low because the number of participants per study was 73.5. Sensitivity analysis (one study removed) yielded pooled estimates of 28% with respect to the original pooled estimate.

### 3.7. Results of the Meta-Analysis in Patients with PHD with Fixed and Non-Fixed Head Condition

**Figure 5:**
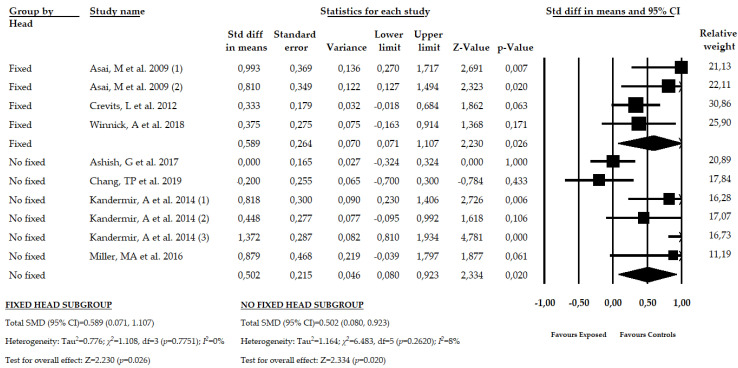
Forest plot for the subgroup analysis in patients with PHD with fixed and non-fixed head condition.

A subgroup analysis was made to assess the misperception of the SVV in function of the head position. Three studies [52,55,57] with four independent comparisons provided 303 participants with PHD with a fixed head position, and four studies [53,54,56,58] with six independent comparisons reported 513 participants with a non-fixed head position to assess the SVV. Our findings show, without heterogeneity, a moderate misperception of VV in PHD patients with a fixed (SMD = 0.589; CI 95% = 0.071, 1.107; *p* = 0.026) and non-fixed head position (SMD = 0.502; CI 95% = 0.080, 0.923; *p* = 0.020) (Table 5, Figure 5, Supplementary Figure S1). Risk of publication bias must be considered in the fixed head subgroup, where the trim-and-fill method suggested a variation of 23% with respect to the original pooled effect (Figure S4). The precision level was low due to the low number of participants per study in each subgroup (75.75 in the fixed head subgroup and 85.5 in the non-fixed head subgroup).

### 3.8. Results of the Meta-Analysis in Patients with PHD Using the Bucket Test or the Rod Projected Screen Test

**Figure 6:**
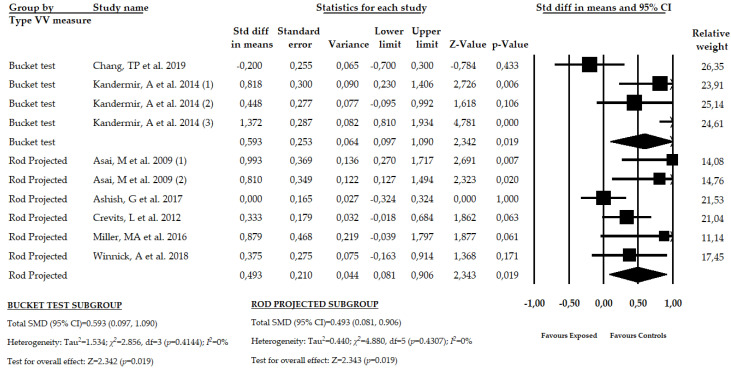
Forest plot for the subgroup analysis in patients with PHD using the bucket test or rod projected test in a dark room to measure the SVV.

A final subgroup analysis was carried out to assess the misperception of SVV in function of the used test. Two studies [54,56] with four independent comparisons reported data of 345 participants with PHD in which the bucket test was used, and five studies [52,53,55,57,58] with six independent comparisons provided data of participants with PHD that used a rod projected test to assess the SVV in a dark room. Our results show a similar moderate misperception of the SVV in patients that used the bucket test (SMD = 0.593; 95% CI = 0.097, 1.090; *p* = 0.019) and in patients that use the rod projected screen test (SMD = 0.493; 95% CI = 0.081, 0.906; *p* = 0.019) (Figure 6, Table 5, Supplementary Figure S1). Heterogeneity was not present in any subgroup and the precision level was low. A high risk of publication bias must be considered in the rod projected subgroups, where a variation of 53% with respect to the original pooled effect was found when the trim-and-fill method was applied (Figure S5).

## 4. Discussion

The present systematic review with meta-analysis attempted to analyze the perception of VV in patients with PHD in comparison to healthy controls. Our results show a misperception of VV in patients with PHD when the SVV test is used to assess the sense of verticality. Both migraine and TTH sufferers present an alteration in verticality perception, but this is more remarkable in patients with TTH, and could be due to the physio-pathological process involved in these disorders.

The main reason for these results possibly reflects the important role that cervical, vestibular, and visual structures play in both the perception of verticality and the physio-pathological processes of primary headaches. A recent study revealed the concomitance of neck pain-generating activities, instability-inducing activities, and primary headaches, where high visual attention-demanding activities are essential in this process [59]. VV misperception in patients with PHD may be facilitated by alterations in some of the three systems involved in forming the pattern of central verticality (vestibular, visual, and somatosensory systems). The SVV test mainly assesses the vestibular contribution [60], and consequently, our results can be interpreted as an alteration of vestibular inputs in patients with headache. However, these three systems present a great physiological intercorrelation [61], and it is impossible to ensure that proprioceptive and visual input impairments do not contribute to the alterations in perception of verticality in these patients.

Patients with TTH are known to present with oculomotor disturbances, including a reduction in the speed of smooth pursuit and saccadic eye movements [13]. These oculomotor disturbances are related to dysfunction in the upper cervical structures [62], which may cause headaches owing to the convergence of cervical and trigeminal afferent fibers at the trigeminal–cervical complex [17]. The integrity of the oculomotor system is essential for correctly estimating verticality, as tilts of the visual vertical are often associated with the components of an ocular tilt reaction (OTR) [63]. The OTR consists of the combined eye movement of torsion and skew deviation that occurs jointly with SVV tilts as a consequence of head tilt. In response to head tilt, an ocular compensatory motor response and adjustment of the SVV in the opposite direction of head tilt occurs, which allows a correct perception of verticality [63]. Thus, oculomotor disturbances that take place in headache sufferers may induce alterations in OTR that could cause verticality misperceptions.

The relationship between neck pain and headache has been widely documented [5,6,7,8]. The upper cervical region is one of the critical structures providing somatosensory information due to the high number of proprioceptive receptors in this area [62]. The somatosensory system plays an important role in PHD physiopathology processes. In this regard, disruptions of the somatosensory temporal discrimination threshold have been observed during migraine attacks [64], distinguishing migraine and TTH as two different clinical entities [65]. In the case of TTH, excitability of deep nociceptors of upper cervical structures triggers headache attacks, contributing to the sensitization process responsible for TTH chronification. Recently, the first meta-analysis has been published that investigates the alteration of the visual perception of verticality in patients with spinal diseases [66]. The results of this study confirm an impairment of the VV perception in spinal pain, highlighting the importance of proprioceptive inputs in order to generate a perception of VV in patients with spinal pain [66]. The degree of importance of upper cervical structures in both PHD physiopathology and the formation of a three-dimensional central pattern of verticality leads us to conclude that misperception of verticality in patients with headache may be motivated by upper cervical dysfunctions.

Vestibular disorders or unsteadiness are other pathologies that frequently appear jointly with headache [16,67]. A link between the trigeminal nucleus and vestibular nuclei may be responsible for these relationships. This link has been demonstrated in rats [68]. In addition, it has been suggested that an imbalance of the vestibular system may be induced by a painful trigeminal stimulation in migraine patients [18], and some authors have proposed that a vestibular disorder should be considered as an integral manifestation of headache and not as two different and concomitant diseases [69]. The function of the vestibular pathways is crucial in SVV perception. Alterations in SVV perception have been observed in patients with peripheral and central vestibular disorders [63]. Moreover, SSV misperception has not only been observed in patients with unilateral and bilateral vestibular dysfunction [70], but the SVV has also been correlated with clinical vertigo symptoms in patients with acute unilateral vestibular neuritis [71]. Thus, vestibular disorders may be the key to verticality misperception in patient with PHD.

Taking into account the importance of the vestibular, visual, and proprioceptive systems, especially in the structures of the neck, both in the development of migraine and in the perception of verticality, the findings of the present review highlight the importance of considering these three systems in the diagnosis and management of PHD. Our review presents some limitations that must be considered. First, some studies found in the bibliographic search were not included in the meta-analysis due to the absence of statistical data of VV perception in both exposed and healthy control groups. Second, the low number of published studies included in our review may explain the possible publication bias found. In addition, the risk of publication bias could not be studied for TTH comparisons. Third, this low number of studies included and the low sample size must be taken into account in the generalization of our results, especially in TTH group, where fewer studies were included and there was a smaller sample size compared to migraines; therefore, these results should be viewed with caution. Fourth, the moderate quality of the studies included may have contributed to the generalization of our findings. The unequal score obtained in the NOS between exposed and non-exposed groups can produce a possible selection bias that must be considered. Another limitation was that no study analyzed the VV perception using the RFT test. Due to this, this study does not assess the visual and somatosensory contribution to the sense of verticality in patients with PHD. In future studies, it will be interesting to assess VV perception trying to differentiate between the three principal systems involved in the formation of the central pattern of the sense of verticality. In patients with PHD, assessing visual and proprioceptive cues using the RFT is particularly suitable due to the importance of the visual and somatosensory systems in the physiopathology processes of migraine and TTH, allowing deeper knowledge of these processes. Another aspect to be highlighted is the variability of the criteria used to diagnose PHD, which can affect the generalizability of the results. Finally, an analysis that evaluated the perception of VV when the subjects were head tilted could not be performed, due to the fact that only one study that assessed VV in this condition met the proposed inclusion criteria, so no conclusion can be drawn from this. However, it is important to highlight this fact and encourage further research on this condition.

## 5. Conclusions

Patients with PHD showed a misperception of VV when, mainly, the vestibular contribution to the sense of verticality was assessed with the SVV test. More specifically, a misperception of VV was found separately in patients with migraine and TTH, showing a higher alteration of the SVV in patients with TTH, although the sample size in each subgroup was small. However, a substantial risk of publication and selection bias needs to be kept in mind when interpreting these results. Future studies should analyze the visual and somatosensory contributions to building the sense of verticality using the RFT in patients with PHD.

## Figures and Tables

**Table 1:** Bibliographic search strategy.

Health Databases	Search Strategy
**Medline PubMed**	(headache disorders, primary[mh] OR headache disorders, primary[tiab] OR headache disorders[mh] OR headache disorders[tiab] OR headache[mh] OR headache[tiab] OR tension-type headache[mh] OR tension-type headache[tiab] OR headache*[tiab] OR migraine disorders[mh] OR migraine disorders[tiab] OR migraine with aura[mh] OR migraine with aura[tiab] OR migraine without aura[mh] OR migraine without aura[tiab] OR “vestibular migraine”[tiab]) AND (perception of verticality[tiab] OR visual vertical*[tiab] OR verticality sense[tiab] OR subjective visual vertical[tiab] OR “svv”[tiab])
**Scopus**	(TITLE-ABS-KEY ((*“migraine”* OR *“migraine disorders”* OR *“vestibular migraine”* OR *“migraine with aura”* OR *“migraine without aura”* OR *“headache disorders”* OR *“headache”* OR *“tension-type headache”*)) AND TITLE-ABS-KEY ((*“perception of verticality”* OR “visual vertical*” OR *“verticality sense”* OR *“subjective visual vertical”*)))
**Web of Science**	TOPIC: ((*migraine* OR *migraine disorders* OR *vestibular migraine* OR *migraine with aura* OR *migraine without aura* OR *headache disorders* OR *headache* OR *tension-type headache* OR *headaches*)) AND TOPIC: ((*perception of verticality* OR *visual verticality* OR *verticality sense* OR *subjective visual vertical*))
**Cinahl**	(MH headache, primary OR AB headache, primary OR MH migraine OR AB migraine OR MH OR AB vestibular migraine OR MH tension headache OR AB tension headache or AB headache) OR (AB perception of verticality OR AB verticality sense OR AB subjective visual vertical OR AB visual vertical)
**Scielo**	(migraine OR vestibular migraine OR headache) AND (verticality OR verticality perception OR verticality sense OR subjective visual vertical)

**Table 2:** Main characteristics of the studies included in the meta-analysis.

				Exposed Group				Healthy Controls		
Author and Year	N Comp	N_S_	N	Mean Age	Sex (M/F)	Type of PHD	Diag. Criteria	N	Mean Age	Sex (M/F)
Asai et al. 2009 [52]	2	53	37	44	12/25	MIG/TTH	IHS	32	44	10/22
Ashish et al. 2017 [53]	1	148	66	39.8	26/40	MIG	Neuh.	82	37.2	52/30
Chang et al. 2019 [54]	1	63	36	43	5/31	MIG	ICVD	27	NR	NR
Crevits et al. 2012 [55]	1	143	47	38	6/41	MIG	IHS	96	39	30/66
Kandemir et al. 2014 [56]	3	94	64	35.5	6/58	MIG/TTH	IHS	30	32.8	5/25
Miller et al. 2016 [58]	1	20	10	33	2/8	MIG	IHS	10	27	6/4
Winnick et al. 2018 [57]	1	54	27	43	8/19	MIG	ICVD	27	41	11/19

Abbreviations: CC = Cases and controls study; F = female; M = male; MIG = migraine; N = number of participants; N comp = number of comparisons of each study; NR = not reported data; N_S_ = sample of each study; PHD = primary headache disorders; Diag = diagnosis; SVV = subjective visual vertical test; TTH = tension-type headache; VVP = perception of visual verticality; HIS = The International Classification of Headache Disorders; ICVD = International Classification of Vestibular Disorders; Neuh = Neuhauster’s Classification.

**Table 3:** Characteristics of the SVV measure in studies included in the review.

Author and Year	SVV Type	Rod Adjustment Method	Room Condition	Body Position	Head Position	Initial Angle	Number of Rep.	Final SVV Value Expressed
Asai et al. 2009 [52]	Rod projected	Potentiometer by rotating handle	Complete darkness	Upright sitting	0º Fixed	Fixed 40°	8	Absolute error mean
Ashish et al. 2017 [53]	Screen rod projected	Joystick handle (potentiometer)	Dark room	Upright sitting	No fixed	Random range 5–20°	6	Absolute error mean
Chang et al. 2019 [54]	Bucket test	Hands in the bucket	Dark bucket	Upright sitting	No fixed	Random	3	Absolute error mean
Crevits et al. 2012 [55]	Screen rod projected	Infrared remote controlled potentiometer	Totally Dark room	Upright sitting	0° fixed	Random	10	Error mean
Kandemir et al. 2014 [56]	Bucket test	Hands in the bucket	Translucent plastic bucket	Upright sitting	No fixed	Random	10	Error mean
Miller et al. 2016 [58]	Screen rod projected	Handle dial	Dark room	Upright sitting	No fixed	Random range ±30°	8	Error mean
Winnick et al. 2018 [57]	Screen rod projected	Handle controller	Dark room	Upright sitting	0°, ±20° fixed	Random	100	Error mean

Abbreviations: Rep = Repetition.

**Table 4:** Newcastle–Ottawa Scale (NOS) score for the methodological quality assessment of included studies.

Study	S1	S2	S3	S4	C1	E1	E2	E3	Total
Asai et al. 2009 [52]	*	*	-	-	**	*	*	-	6
Ashish et al. 2017 [53]	*	*	-	*	**	*	*	-	7
Chang et al. 2019 [54]	*	*	-	-	**	*	*	-	6
Crevits et al. 2012 [55]	*	*	-	-	**	*	*	-	6
Kandemir et al. 2014 [56]	*	*	-	*	**	*	*	-	7
Miller et al. 2016 [58]	*	*	-	-	**	*	*	-	6
Winnick 2018 [57]	*	*	-	*	**	*	*	-	7

Each study can be awarded a maximum of one star for each numbered item within the Selection (S) and Exposure (E) categories. A maximum of two stars can be given for Comparability (C). S1 = Adequate case definition; S2 = representativeness of the cases; S3 = selection of controls; S4 = definition of controls; C1 = comparability of cases and controls; E1 = ascertainment of exposure; E2 = same method of ascertainment for cases and controls; E3 = non-response rate.

**Table 5:** Summary of findings in the meta-analyses.

					Effect Size			Publication Bias			Heterogeneity		
Groups		K	N	N_s_	SMD	95% CI	*p*	Funnel Plot	Trim-and-Fill		*q*-Value	*I* ^2^	*p*
									Adj SMD	% of Var			
Overall PHD		10	816	81.6	0.530	[0.225, 0.836]	<0.001	Slight asym	0.335	37%	8.779	0%	0.458
PHD subgroups	MIG	8	669	83.7	0.369	[0.1, 0.638]	0.007	Slight asym	0.267	28%	7.481	6.5%	0.38
	TTH	2	147	73.5	1.122	[0.540, 1.704]	<0.001	-	-	-	1	0%	0.317
Head position	Fixed	4	303	75.75	0.589	[0.071, 1.107]	0.026	Slight asym	0.45	23%	1.108	0%	0.7751
	No fixed	6	513	85.5	0.502	[0.080, 0.923]	0.020	Sym	0.502	0%	6.483	8%	0.2620
Type SVV measure	Rod Projected	6	471	78.5	0.493	[0.081, 0.906]	0.019	Asym	0.21	53%	4.880	0%	0.4307
	Bucket test	4	345	86.25	0.593	[0.097, 1.090]	0.019	Sym	0.593	0%	2.856	0%	0.4144

Abbreviations: Asym = Asymmetric; *I*^2^ = degree of inconsistence; K = number of studies; N = number of participants in each meta-analysis; N_s_ = number of participants per study; *p* = *p*-value; Adj = adjusted; % of var = percentage of variation; PHD = primary headache disorder; SMD = standardized mean difference; TTH = tension-type headache; 95% CI = 95% confidence interval; Asym = asymmetric; Sym = symmetric.

## Supplementary Materials

The following are available online at https://www.mdpi.com/2076-3425/10/10/664/s1, Figure S1: Bar chart of SVV mean value on PHD patients and healthy controls in each assessed condition; Figure S2: Funnel plot for SVV test in overall PHD patients; Figure S3: Funnel plot for SVV test in migraine patients; Figure S4: Funnel plot for SVV test in fixed head subgroup; Figure S5: Funnel plot for SVV for rod projected test subgroup.
