# Supplementary material for: Misperception of Visual Verticality in Patients with Primary Headache Disorders: A Systematic Review with Meta-Analysis
Source: Brain Sci. 2020 Sep 24;10(10):664. doi: 10.3390/brainsci10100664 (PMC7598580; doi:10.3390/brainsci10100664)
Supplement: Supplementary file 1 [file brainsci-10-00664-s001.zip › brainsci-909694-Sup-2/Figure S1. Bar chart of SVV mean value on PHD patients and healthy controls in each assessed condition.docx]

**Figure S1:** Bar Chart of SVV Mean Value in PHD patients and healthy controls in each condition assessed

|  | **PHD Patients** | **Healthy Controls** |
| --- | --- | --- |
| **Overall** | 1,0074 | 0,39 |
| **Migraine** | 0,91 | 0,41 |
| **TTH** | 1,15 | 0,2 |
| **Fixed Head** | 1,04 | 0,25 |
| **Non fixed Head** | 0,97 | 0,44 |
| **Bucket test** | 0,86 | 0,225 |
| **Rod projected** | 1,08 | 0,5 |
