# Supplementary material for: Misperception of Visual Verticality in Patients with Primary Headache Disorders: A Systematic Review with Meta-Analysis
Source: Brain Sci. 2020 Sep 24;10(10):664. doi: 10.3390/brainsci10100664 (PMC7598580; doi:10.3390/brainsci10100664)
Supplement: Supplementary file 1 [file brainsci-10-00664-s001.zip › brainsci-909694-Sup-2/Table S1. Collected mean SVV value on PHD patients and healthy controls.docx]

|  | **PATIENTS WITH PHD** | | | **HEALTHY SUBJECTS** | | |
| --- | --- | --- | --- | --- | --- | --- |
|  |  | **SVV Measure** | |  | **SVV Measure** | |
| **STUDY** | **N** | **MEAN** | **SD** | **N** | **MEAN** | **SD** |
| Asai, M et al. 2009 (1) | 17 | 1,5 | 1,2 | 16 | 0,6 | 0,4 |
| Asai, M et al. 2009 (2) | 20 | 1,3 | 1,1 | 16 | 0,6 | 0,4 |
| Ashish, G et al. 2017 | 66 | 1,5 | 0,9 | 82 | 1,5 | 0,7 |
| Chang, TP et al. 2019 | 36 | 0,7 | 1 | 27 | 0,9 | 1 |
| Crevits, L et al. 2012 | 47 | 0,4 | 1,43 | 96 | 0,01 | 1,02 |
| Kandermir, A et al. 2014 (1) | 20 | 1 | 1,5 | 30 | 0 | 1 |
| Kandermir, A et al. 2014 (2) | 24 | 0,75 | 2,25 | 30 | 0 | 1 |
| Kandermir, A et al. 2014 (3) | 30 | 1 | 0,25 | 30 | 0 | 1 |
| Miller, MA et al. 2016 | 10 | 0,884 | 0,788 | 10 | 0,097 | 0,991 |
| Winnick, A et al. 2018 | 27 | 1,04 | 2,23 | 27 | 0,25 | 1,97 |
|  | **297** | **1,0074** | **0,3522** | **364** | **0,39** | **0,5** |

**Table S1.** Collected mean SVV value on PHD patients and healthy controls
