# Supplementary figures and images for: Misperception of Visual Verticality in Patients with Primary Headache Disorders: A Systematic Review with Meta-Analysis
Source: Brain Sci. 2020 Sep 24;10(10):664. doi: 10.3390/brainsci10100664 (PMC7598580; doi:10.3390/brainsci10100664)

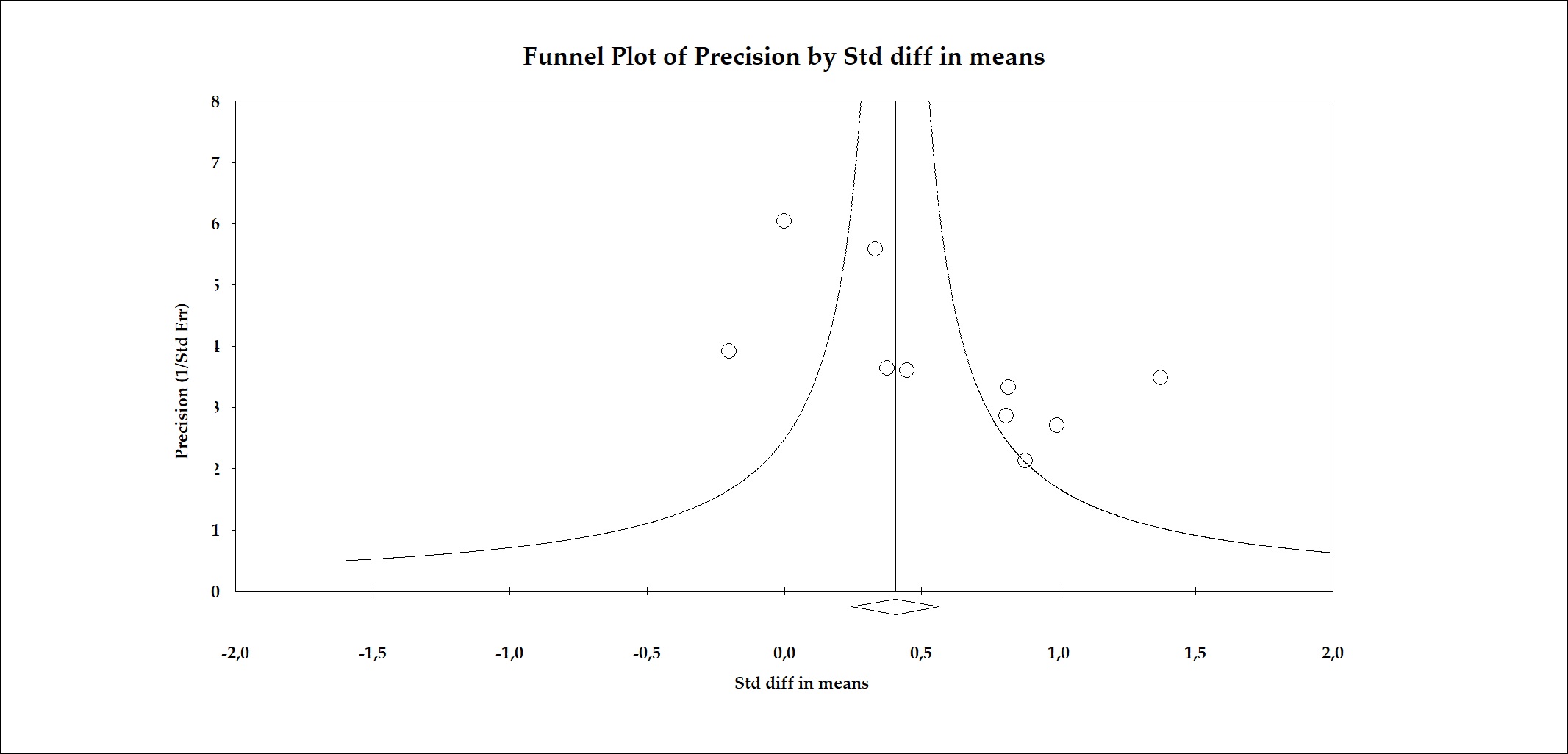

Supplement: Supplementary file 1 [file brainsci-10-00664-s001.zip › brainsci-909694-Sup-2/Figure S2. Funnel plot for SVV test in overall PHD patients_.jpg]

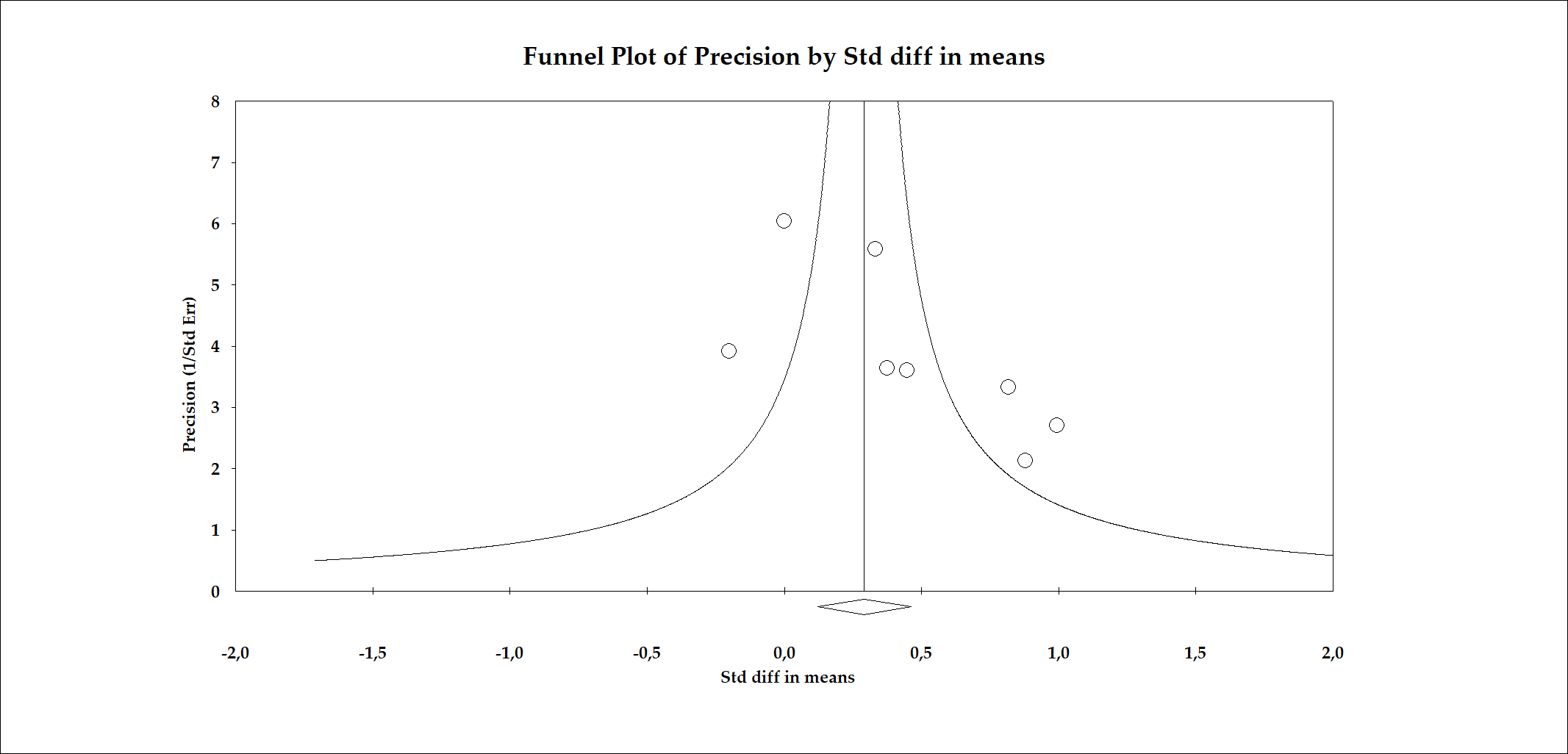

Supplement: Supplementary file 1 [file brainsci-10-00664-s001.zip › brainsci-909694-Sup-2/Figure S3. Funnel plot for SVV test in migraine patients_.tif]

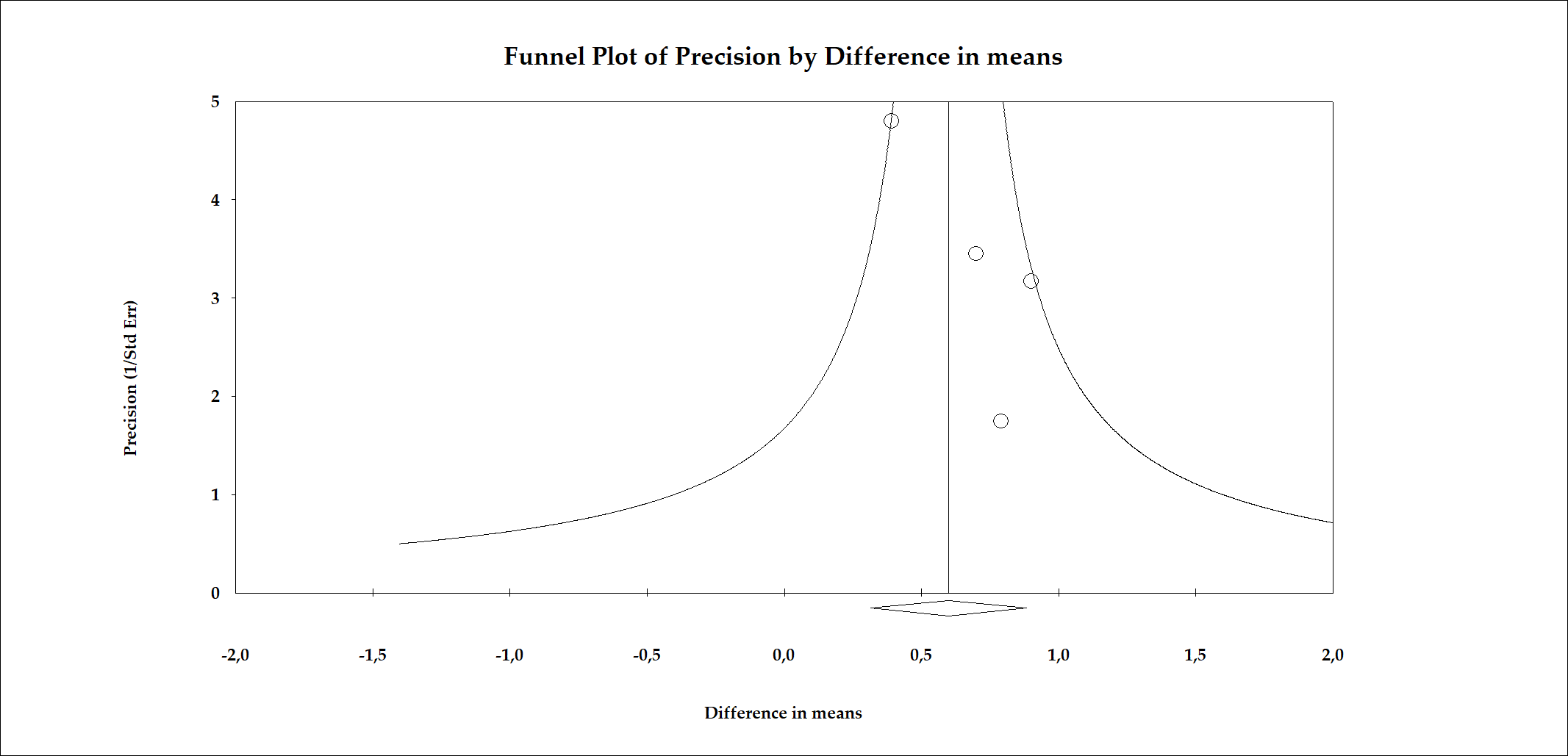

Supplement: Supplementary file 1 [file brainsci-10-00664-s001.zip › brainsci-909694-Sup-2/Figure S4. Funnel plot for fixed head subgroup.tif]

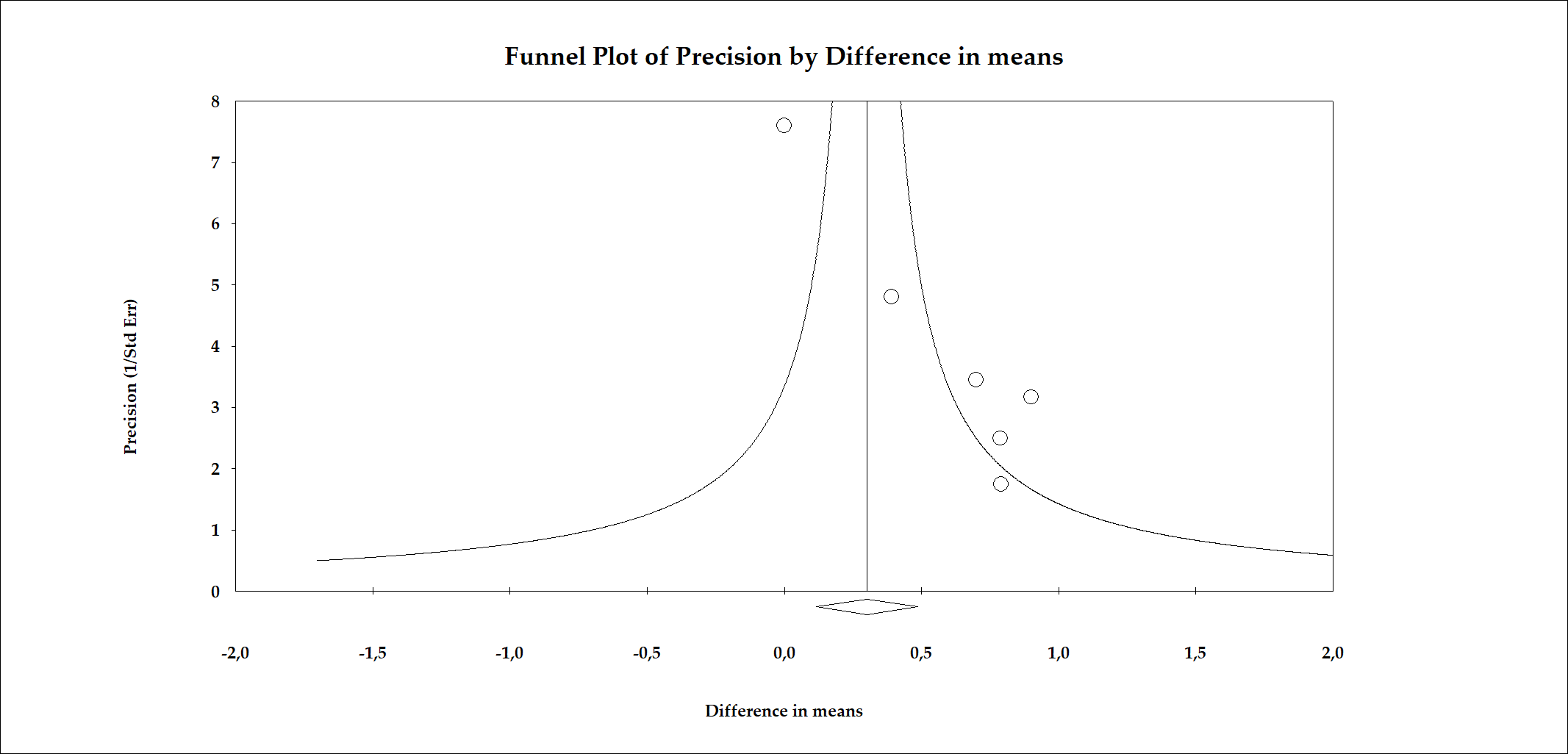

Supplement: Supplementary file 1 [file brainsci-10-00664-s001.zip › brainsci-909694-Sup-2/Figure S5. Funnel Plot for rod projected test subgroup.tif]
